# Conjugated linoleic acid (CLA)-induced milk fat depression: application of RNA-Seq technology to elucidate mammary gene regulation in dairy ewes

**DOI:** 10.1038/s41598-019-40881-3

**Published:** 2019-03-14

**Authors:** Aroa Suárez-Vega, Beatriz Gutiérrez-Gil, Pablo G. Toral, Gonzalo Hervás, Juan José Arranz, Pilar Frutos

**Affiliations:** 1 Departamento de Producción Animal, Facultad de Veterinaria, Universidad de León, Campus de Vegazana s/n, León, 24071 Spain; 2 Instituto de Ganadería de Montaña (CSIC-ULE), Finca Marzanas s/n, Grulleros, 24346 León, Spain

## Abstract

Milk fat depression (MFD) is characterized by a reduction in the content of milk fat, presumably caused by the anti-lipogenic effects of rumen biohydrogenation intermediates, such as *trans*-10 *cis*-12 conjugated linoleic acid (CLA). In this study, RNA-Seq technology was used to help elucidate the mammary responses involved in CLA-induced MFD in lactating ewes. To this end, we compared the milk somatic cell transcriptome of ewes suffering from CLA-induced MFD with control ewes (i.e., those without MFD), as well as with ewes fed a diet supplemented with fish oil (FO-MFD) that we previously reported affects the mammary transcriptome. In the differential expression analysis between CLA-MFD and controls, we identified 1,524 differentially expressed genes (DEGs), whereas 653 were detected between CLA- and FO-MFD groups. Although this article focuses on lipid metabolism, CLA affected the expression of many genes related to other biological processes, especially immunity. Among the 55 genes shared by both MFD conditions, some genes linked to fatty acid synthesis, such as *ACACA*, *AACS*, *ACSS2*, or *ACSS3*, were downregulated. In addition, this study provides a list of candidate genes that are not usually considered in the nutrigenomics of MFD but that may act as key regulators of this syndrome in dairy ewes.

## Introduction

Milk fat depression (MFD) syndrome is generally characterized by a strong reduction in milk fat content without significant changes in the yield of milk or other milk components^1^. This syndrome was first described in dairy cows fed highly fermentable or plant oil-containing diets, where it is primarily mediated by the rumen metabolite *trans*-10 *cis*-12 CLA^2^. Dairy sheep and goats are not affected by those feeding conditions, but they may suffer from the syndrome when their diets are supplemented with marine lipids^3,4^, resulting in economic losses because most ovine and caprine milk is destined for cheese manufacturing.

Diet supplementation with marine lipids (e.g., fish oil (FO) or marine algae) is a nutritional strategy that aims at modulating milk fatty acid (FA) composition towards a potentially healthier profile for consumers (e.g., with increased concentration of CLA^5,6^). Reported health benefits of CLA include anti-obesity, anti-carcinogenic, anti-atherogenic and immunomodulatory effects^7^.

Although marine lipid-induced MFD is not due to greater ruminal accumulation of the anti-lipogenic *trans*-10 *cis*-12 CLA, mammary lipogenesis is also inhibited in small ruminants receiving this isomer from an external source^4,8,9^. In fact, dairy ewes displaying *trans*-10 *cis*-12 CLA-induced MFD have been proposed as a model to examine mammary mechanisms explaining low-fat milk syndrome in cows^9^. Nevertheless, these ewes might not be a good model to study the diet-induced MFD in ewes themselves, which is caused by marine lipid supplementation.

In a study conducted in lactating sheep fed a diet supplemented with either FO or *trans-*10 *cis-*12 CLA^4^, similar changes were observed in the milk fat content and in the processes of *de novo* FA synthesis and FA uptake, suggesting that both types of MFD might share regulatory mechanisms. However, milk FA composition differed significantly between these two types of MFD and supported that FO-induced MFD was not mediated by *trans-*10 *cis-*12 CLA but likely by other potentially anti-lipogenic rumen FAs. The ability of these latter metabolites to downregulate the expression of key mammary lipogenic genes, as demonstrated for *trans-*10 *cis-*12 CLA^9^, is unkown. In another study examining dairy ewes fed FO or *trans-*10 *cis-*12 CLA^10^, differences in the mRNA abundance of certain candidate genes involved in mammary lipogenesis indicated substantial divergence in the transcriptional mechanisms underlying FO- or CLA-induced MFD. Because mechanisms driving the response to marine lipids are poorly understood, especially in sheep, we used RNA sequencing technology (RNA-Seq) to provide a comprehensive profile of the transcriptomic changes occurring in the mammary gland of ewes suffering from FO-induced MFD^11^. This technology has been demonstrated as a useful approach to identify target genes in nutrigenomics, as well as to understand the biological processes underlying MFD in other ruminants^12,13^.

The objective of this study was to help characterize the mammary responses involved in CLA-induced MFD in lactating ewes by evaluating changes in the milk somatic cell transcriptome caused by dietary inclusion of a rumen-protected product rich in *trans*-10 *cis*-12 CLA. Comparisons were conducted not only with control ewes fed the same diet without lipid supplementation (i.e., in the absence of MFD, control) but also with ewes receiving a diet supplemented with FO that we previously reported^11^ to cause shifts in mammary gene expression.

## Materials and Methods

### Animals and diets

All protocols animals were approved by the Animal Welfare Committee of the Instituto de Ganadería de Montaña, the Spanish National Research Council (CSIC) and the Junta de Castilla y León (Spain), following proceedings described in Spanish and EU legislations (R.D. 53/2013, and Council Directive 2010/63/EU). All animals used in this study were handled in strict accordance with good clinical practices, and all efforts were made to minimize suffering. This study is an integral part of a larger experiment conducted to investigate the low-fat milk syndrome induced by either marine lipids or *trans*-10, *cis*-12 CLA in dairy ewes. Three groups of dairy ewes were submitted to the following treatments: control (no MFD), FO-induced MFD, and CLA-induced MFD. In a first report^10^, treatment effects on the mRNA abundance of candidate lipogenic genes in milk somatic cells (MSCs) were analyzed by RT-qPCR. However, given the suggested differences in the transcriptional mechanisms between MFD conditions, we decided to go further in this research by using RNA-seq technology. Because of the complexity of this latter analysis, the study was split into two works: the first one addressed the condition caused by dietary marine lipids and was published by Suárez-Vega *et al*.^11^. This work, the second one, deals with CLA-induced MFD.

Briefly, lactating Assaf ewes at mid lactation (66 ± 1.8 days in milk at the beginning of the assay; parity = 2.5 ± 0.52) were individually housed and randomly divided into two groups that were assigned to one of two dietary treatments: no lipid supplementation (control) or supplementation with 10 g of a rumen-protected CLA product/kg of diet dry matter (CLA-MFD). The commercial CLA supplement (Lutrell Pure, BASF, Ludwigshafen, Germany) contained similar amounts of *cis*-9 *trans*-11 and *trans*-10 *cis*-12 isomers. The basal diet consisted of a total mixed ration based on alfalfa hay and concentrates (forage:concentrate ratio 40:60). All animals were fed the control diet for a 21-d adaptation period and then both experimental diets for 40 more days. Samples were collected at the end of the experiment, when ewes on the CLA-MFD treatment exhibited a stable decrease in milk fat concentration (monitored daily by infrared spectrophotometric analysis of raw milk samples; ISO 9622:1999). Diets, which were prepared weekly and included molasses to reduce selection of components, were offered *ad libitum*, and animals were milked twice daily in a single side milking parlor (DeLaval, Madrid, Spain). Further details can be found in Toral *et al*.^10^.

Although this trial was conducted with a higher number of sheep, based on criteria of maximum cost of the experiment and selection of phenotypically homogenous individuals, RNA-Seq analyses were initially performed on four ewes per group. Nevertheless, due to the high variability of data within the CLA-MFD condition (details are explained below), two more individuals were included in this treatment group (n = 6).

Consumption of dietary *trans*-10 *cis*-12 CLA significantly reduced milk fat concentration in ewes (5.99 vs. 3.76%, SED = 0.399, for control and CLA-MFD, respectively). This decrease (37.2%) was stronger than that observed in FO-MFD animals^11^. Milk fat secretion was reduced to a similar extent (−33.5% relative to control; *P* < 0.001). On the other hand, as expected, feed intake (on average, 2.70 kg/d), milk production (on average, 1.46 kg/d), and milk composition in terms of protein, lactose, somatic cell count (log_10_) and FA profile did not significantly differ compared to control. Milk somatic cell count was similar in MFD- and control ewes (*P* > 0.10), but it was 5.6% lower in FO- than in CLA-MFD treatment (*P* = 0.012). Mean values remained low in the three dietary conditions (mean of non-transformed data: 77 × 10^3^/mL of raw milk).

### Sampling, RNA extraction and sequencing

Milk samples for RNA extraction were collected on days 38 and 39 on treatments. As detailed by Suárez-Vega *et al*.^14^, the sample collection was performed one hour after milking and ten minutes after the injection of 5 IU of Oxitocine Facilpart (Syva, León, Spain) to maximize the concentration of MSCs. Udders were cleaned with water and soap; then, they were disinfected with povidone iodine; and finally the nipples were washed with RNAseZap (Ambion, Austin, TX, USA). To ensure high yield and quality of RNA, samples were obtained by hand-milking each half of the mammary gland of ewes into an RNAse-free 50-mL tube (2 samples/ewe). A sterile gauze was used to cover the tube and filter the milk. Samples were held in ice and transferred immediately to the laboratory where they were processed.

For the RNA extraction, MSCs in the 50 ml of fresh milk were pelleted by centrifugation at 650 × *g* for 10 min at 4 °C in the presence of a final concentration of 0.5 mM of EDTA. The cell pellet was washed with 10 mL of PBS (pH 7.2 and 0.5 mM of EDTA) followed by an additional centrifugation at 650 × *g* for 10 min at 4 °C. Washing and centrifugation procedures were repeated twice using 2 and 1.5 mL of the same PBS solution. Then, total RNA was extracted and purified from the milk cell pellet with 500 μL of TRIzol (Invitrogen, Carlsbad, CA, USA).

Once RNA was extracted, an Agilent 2100 Bioanalyzer device (Agilent Technologies, Santa Clara, CA, USA) was used to assess RNA integrity value (RIN). The mean RIN of the CLA-MFD RNA samples was 7.8 ± 0.22 (range 6.8–8.4).

RNA sequencing was conducted at CNAG (Centro Nacional de Análisis Genómico, Barcelona, Spain), where the TruSeq Stranded Total RNA Library Prep Kit (Illumina, San Diego, CA, USA) was used to generate stranded paired-end libraries with 300 bp fragments. The fragments were sequenced to a minimum depth of 30 million reads on an Illumina Hi-Seq 2000 sequencer (Fasteris SA, Plan-les-Ouates, Switzerland), generating stranded paired-end reads of 75 bp. CLA-MFD samples were sequenced in two different batches: four samples together with four FO-MFD and four control samples, while the other two samples were sequenced at a later stage.

### Power calculations

Power calculations were performed using the online tool Scotty (http://scotty.genetics.utah.edu/scottyOutput.php). A table of counts with MSC transcriptome gene expression for four control and four CLA-MFD samples was used as input to estimate the power of the differential expression analyses. Moreover, the following criteria were set: an alignment rate of 75%, a maximum of six replicates per condition, a cost per replicate of 100 (control) and 100 (test) US Dollars (USD), a read depth between 10–50 million reads, a cost per million reads aligned to genes of 100 USD, a maximum cost of the experiment of 100,000 USD, 50% of differentially expressed genes detected with a fold change of two, a p-value cut-off of 0.05, and a minimum of 50% of genes with at least 50% maximum power.

### Alignment and quantification

Alignment, quantification, differential expression analysis and functional annotation were performed using the RNA-Seq data extracted from the CLA-MFD samples described above but also using controls and FO-MFD data detailed by Súarez-Vega *et al*.^11^.

Samples were aligned to the Oar_v.3.1 ovine reference genome using the Oar_v.3.1_r88 annotation, and all the files were downloaded from Ensembl database. Sample alignment was performed using STAR v.2.4.0^15^. Within the basic command line, we used the options “–*outFilterType BySJout*” to reduce spurious junctions, “–*outWigStrand Stranted*” to indicate that our RNA-Seq data was stranded, and the option “*–quantMode TranscriptomeSAM*” to create an output file with alignments translated into transcript coordinates required by the quantification tool RSEM^16^. The quantification step was performed using RSEM v.1.3.0^16^ with the options*–bam* and*–no-bam-output* to indicate that a bam file with reads aligned to the transcriptome was provided as an input and should not be created by RSEM. The options*–paired-end* and*–forward-prob 0* were used to indicate that our RNA-Seq data is paired-end and stranded, with the upstream read derived from the reverse strand. Moreover, we applied the options*–estimate-rspd* to estimate the read start position distribution,*–calc-ci* to calculate 95% credibility intervals and posterior mean estimates and*–seed 12345* to set the seed for the random number generators used in calculating posterior mean estimates and credibility intervals.

### Differential expression analysis

Data from RSEM was imported to the R environment with the R package tximport^17^. The program DESeq.2 v.1.18.1^18^ was used to perform differential expression analysis. For the analysis, technical replicates from the same sample were first collapsed with the function *collapseReplicates*. Based on the rlog transformed gene counts, we performed a principal component analysis (PCA) to cluster the samples based on gene expression data.

**Figure 1:**
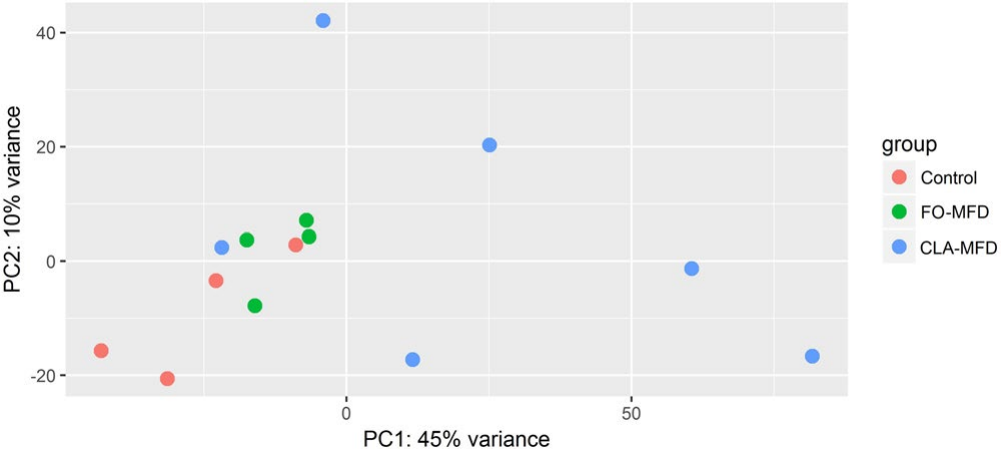
Principal Component Analysis (PCA) plot of milk somatic cell transcriptomes from control (red), CLA-induced (CLA-MFD; blue), and fish oil-induced milk fat depression (FO-MFD; green) ewes.

Due to the high variability of data within the CLA-MFD group in the estimation of dispersion of the power analysis and in the PCA plot performed with R (Fig. 1), we decided to perform the comparison between different conditions separately. Thus, the function *DESeq*, which estimates size factors, dispersion, and performs differential expression analysis, was run pairwise only with the samples from the two conditions to be compared (CLA-MFD vs. control, and CLA-MFD vs. FO-MFD, as recommended by DESeq2 when there is an extreme range of within-group variability).

For CLA-MFD samples, we first evaluated batch effect through a likelihood ratio test (lrt) to compare total model with the model only, evaluating the batch effect and identifying the genes whose expression level was influenced by the batch. Next, differentially expressed genes (DEGs) between the different conditions (CLA-MFD vs. control, and CLA-MFD vs. FO-MFD) were identified using a Wald test. Genes with a p-adjusted value (padj) <0.05 after the correction of p-values for multiple testing using the Benjamini–Hochberg’s approach were considered DEGs.

### Functional enrichment analysis

Several Gene Ontology (GO) term functional enrichment analyses were performed using WebGestaltR^19^ with the following options: *minNum* (minimum number of genes per category) = 5 of the total genes in the input, *fdrMethod* (False discovery rate method) = BH (Benjamini–Hochberg), *sigMethod* (Significant method) = FDR (False Discovery Rate), and *fdrThr (FDR threshold)* = *0*.*05*. These analyses were performed for the different lists of DEGs highlighted by the previous analyses: upregulated or downregulated genes identified by different comparisons performed for the CLA-MFD samples, as well as genes usually identified as DEGs after comparing the two MFD treatments (CLA-MFD and FO-MFD) versus control.

## Results

### Power of the design

Results for the power analysis showed that the most cost-effective experiment for performing the differential expression analysis with sufficient power to detect at least 50% of the genes at a p-value cut-off of 0.05 and a true fold change of two included four replicates sequenced to a depth of 10 million reads aligned to genes per replicate. The most powerful experiment was sequencing six replicates to a depth of 50 million reads aligned to genes per replicate. CLA replicates used to test the power showed a dispersion of 0.39, whereas the estimated dispersion of control samples was 0.20, which are within the common values of over dispersion of biological replicate pairs (0.2–0.4; http://scotty.genetics.utah.edu/). Thus, given the dispersion of the CLA-MFD samples, we decided to sequence six rather than four samples to increase the power of our experiment. The experimental conditions of the study presented herein meet the power requirements to detect an acceptable proportion (approximately 60%) of differentially expressed genes.

### Sequencing and alignment: basic statistics

Samples were sequenced to a minimum of 30 million paired-end reads. However, there were one control and two CLA-MFD samples that did not reach the minimum reads fixed and were resequenced. For the majority of samples, which reached the minimum depth fixed, the average paired-end reads generated per sample was 41,317,509 (SD = 5,239,094.7). For a control sheep, two technical replicates were sequenced with 17,686,309 and 24,006,681 reads per replicate. The two CLA-MFD samples that did not reach the minimum depth fixed initially were sequenced twice, with 19,597,009 and 22,390,871, and 17,686,309 and 24,006,681 paired end reads, respectively. All fastq files obtained from sequencing were individually mapped to the sheep reference genome (Oar_v.3.1). The average alignment rate of the individual samples to unique sites in the sheep genome was 89.9% (SD = 1.35).

### PCA results

Results from the PCA performed based on the rlog-transformed gene counts showed that the first two principal components accounted for 45% (PC1) and 10% (PC2) of the variance (Fig. 1). PC1 seemed to group the samples by conditions and reflected a high level of dispersion for the CLA-MFD samples, whereas FO-MFD and control groups exhibited lower variability.

### Differential expression analysis

#### CLA-MFD vs. Control

Seven genes were affected by the batch, with a padj <0.05 in CLA-MFD samples (*ENSOARG00000000222*, *RND2*, *CCL28*, *ENSOARG00000009395*, *RXFP2*, *ENSOARG00000014187*, *ENSOARG00000015539*). In the differential expression analysis between CLA-MFD and control samples, we found 1,257 upregulated and 271 downregulated genes in CLA-MFD sheep. Four of the genes influenced by the batch (i.e., *ENSOARG00000000222*, *RND2*, *CCL28*, *ENSOARG00000009395*) were identified as differentially expressed in this analysis (specifically, one was upregulated and three downregulated) and were excluded from the list of DEGs used to perform the functional analysis. The 1,256 genes upregulated in the CLA-MFD group were clustered in 1,205 GO terms [994 in the GO biological process category (GO-BP), 95 in the GO Molecular Function category (GO-MF) and 116 in the GO Cellular Component category (GO-CC); Supplementary Table S1]. In the GO-BP, there were 18 terms with an FDR = 0, the majority of which were related to immune response. The highest enriched GO terms within the GO-MF was “*cytokine binding*” (FDR = 4.74E-08) and within the GO-CC was “*endocytic vesicle*”, “*vacuolar part*” and “*side of membrane*” (FDR = 0). Some terms with the highest number of genes linked to lipid metabolism were “*lipid catabolic process*” (29 genes; FDR = 0.01), “*lipid transport*” (28 genes; FDR = 0.04) and “*lipid storage*” (16 genes; FDR = 8.68E-06).

The 268 genes downregulated in the CLA-MFD condition were clustered in 87 GO terms (44 in GO-BP, 19 in GO-MF, and 24 in GO-CC; Supplementary Table S2). The highest enriched terms for each of these categories were “*citrate metabolic process*” (FDR = 1.00E-06), “*cofactor binding*” (FDR = 1.29E-07) and “*mitochondrial matrix*” (FDR = 5.65E-07), respectively. Specific terms directly related to fat metabolism were “*fatty acid metabolic process*” (16 genes; FDR = 2.57E-03), “*fatty acid catabolic process*” (7 genes; FDR = 2.52E-02) and “*fatty-acyl-CoA binding*” (4 genes; FDR = 3.14E-02).

#### CLA-MFD vs. FO-MFD

First, we evaluated the influence of the batch, detecting seven differentially expressed genes due to this effect (*ATP8*, *ENSOARG00000009152*, *ENSOARG00000009395*, *RXFP2*, *ENSOARG00000015539*, *PPIC* and *ENSOARG00000020879*). In the comparison between CLA-MFD and FO-MFD sheep, 654 DEGs were identified (569 upregulated and 85 downregulated in CLA-MFD). Only the *PPIC* gene was differentially expressed both due to the batch effect and between CLA-MFD and FO-MFD; therefore, it was deleted from the list of DEGs to perform the GO analysis. The upregulated genes within CLA-MFD were clustered in 662 GO terms (566 terms within GO-BP, 32 in GO-MF and 64 in GO-CC; Supplementary Table S3). There were four terms in GO-BP with a FDR = 0, all of which related to immunity. The highest enriched terms in GO-MF were “*cytokine receptor activity*” and “*cytokine binding*” (FDR = 7.28E-05), and in the GO-CC “*membrane raft*” and “*membrane microdomain*” (FDR = 2.15E-10). Among the enriched GO terms in CLA-MFD upregulated genes, we found the terms “*lipid storage*” (9 genes; FDR = 8.68E-04), “*regulation of lipid storage*” (7 genes; FDR = 2.07E-03), “*positive regulation of lipid storage*” (4 genes; FDR = 2.65E-02), “*response to fatty acid*” (7 genes; FDR = 4.69E-02), and “*lipid particle*” (6 genes; FDR = 1.29E-02).

Thirty-two significant GO terms (16 in GO-BP, 5 in GO-MF and 11 in GO-CC) were identified when clustering the genes downregulated in the CLA-MFD group (Supplementary Table S4). The highest enriched term in the GO-BP was “*small molecule catabolic process*” (FDR = 1.64E-04), and remarkably, within this category, the majority of terms were related to catabolic processes on lipids/fatty acids. In the remaining categories, GO-MF and GO-CC, the highest enriched terms were “*cofactor binding*” (FDR = 1.97E-05) and “*peroxisome*” (FDR = 3.98E-04).

#### Comparison between CLA-MFD vs. Control and FO-MFD vs. Control

Because MFD is the phenotype shared by CLA-MFD and FO-MFD sheep when compared to the control, DEGs in CLA-MFD vs. control and FO-MFD vs. control were contrasted to assess the similarities between both types of MFD. Gene expression changes caused by FO-induced MFD were detailed in a previous study^11^. However, RNA-Seq bioinformatics analyses have now been repeated with the workflow described above (STAR v.2.4.0, RSEM v.1.3.0 and DESeq.2 v.1.18.1 software) and the new release of the sheep genome assembly annotation (Oar_v.3.1-r88). The number of DEGs identified in the present study in the comparison of FO-MFD vs. control was 237. Among these DEGs, 178 genes were concordant with the 213 DEGs genes described previously^11^; therefore, they were used for the comparison of CLA-MFD vs. control DEGs.

Among upregulated genes in both MFD conditions, we found 35 shared upregulated genes, whereas only 63 were upregulated in FO-MFD, and 1,222 genes were upregulated in CLA-MFD. Concerning downregulated genes, there were 20 genes shared in both MFD conditions, whereas only 60 genes were downregulated in FO-MFD and only 251 in CLA-MFD.

When clustering the 35 common upregulated genes identified for the two MFD conditions, one GO-BP enriched term was identified, “*divalent metal ion export*” (FDR = 3.63E-02). The 20 genes downregulated in both conditions were clustered in 10 significant enriched GO terms (5 GO-BP and 5 GO-MF); the highest enriched terms were “*acyl-CoA metabolic process*” (FDR = 8.40E-03) within the GO-BP category and “*acid-thiol ligase activity*” (FDR = 2.79E-05) within the GO-MF (Supplementary Table S5).

## Discussion

This study was conducted to assess changes in the milk somatic cell transcriptome in dairy sheep displaying CLA-induced MFD to help characterize this condition.

First, the large number of DEGs between CLA-MFD and control sheep stands out (1,524 genes), illustrating that diet supplementation with CLA has a higher impact on the mammary gland transcriptome of dairy ewes than does supplementation with FO (213 DEGs) previously detailed in Suárez-Vega *et al*.^11^. Moreover, considering the global gene expression levels in MSCs (Fig. 1), the observed variability within the CLA-MFD group was much higher than within the other two groups (FO-MFD and control).

A large number of enriched terms related with immune function were identified in the mammary transcriptome of CLA-MFD ewes. Milk somatic cell counts are sensitive to animal health status^20^, but values in this treatment, low and not different from the control, would not explain the observed results. However, the CLA product contained the *trans*-10 *cis*-12 and *cis*-9 *trans*-11 CLA isomers, which have been reported to exert beneficial effects on immunity due to the modulation of T-cell and cytokine responses, as well as activation of the PPARG signaling pathway (reviewed by Viladomiu *et al*., 2016)^21^. This transcription factor, *PPARG*, also seems to have a key role in milk fat synthesis in ruminants, but its implication in CLA-induced MFD, if any, are uncertain^22,23^. Upregulation of *PPARG* in the CLA-MFD samples, which is consistent with qPCR results reported previously as part of the same study^10^, may suggest activation of PPARG-mediated anti-inflammatory mechanisms^21,22^. Among the vast number of terms related to the immune response in our results, contradictory terms linked to both innate and adaptive responses were detected, such as *cytokine activation* and *negative regulation of cytokine production* or *T-cell activation* and *negative regulation of T-cell activation*. This is in line with the controversy concerning the pro-/anti- inflammatory effect of CLA under different environment conditions^21^. Moreover, we speculate that the activation of signaling pathways, such as Toll-like Receptors (TLRs) signaling pathway, and the involvement of other transcription factors upregulated in this study, like nuclear factor kappa B and activator protein-1, could contribute to explain the large transcriptional response seen in sheep MSCs supplemented with CLA. In any event, despite the relevance of the influence of CLA diet supplementation on immunity, dedicated research is required to gain new insight in this regard. Moreover, the subject is outside the framework of this article and therefore will not be further discussed herein.

In this study, we focused our attention on terms involved in lipid metabolism, to try to identify those most directly related to the mechanisms underlying both types of milk fat depression.

Starting with the upregulated genes, in the CLA-MFD vs. control comparison, there were several terms putatively linked to MFD, such as “*negative regulation of lipid storage*”, “*regulation of lipase activity*”, “*lipid catabolic process*”, “*response to fatty acid*” and “*lipase activity*”, suggesting the involvement of CLA in lipolysis in the mammary gland, as recently suggested in the adipose tissue of cows^24^. Thus, upregulated genes within these terms could be postulated as key factors in the MFD caused by CLA. For instance, *carnitine palmitoyltransferase 1* (*CTP1A*), one of the 57 genes clustered in the five GO terms listed above, was reported to be upregulated in goat mammary epithelial cells incubated with CLA^13^. This protein has a pivotal role in the regulation of long-chain fatty acid oxidation in the mitochondria, which might indicate increased energy expenditure in the mammary gland of ruminants in response to CLA supplementation^13^, contrary to what normally occurs during lactation, when fatty acid synthesis predominates over oxidation^25^.

However, other DEGs that were also clustered into terms related to fatty acid oxidation and lipid catabolic processes, *ECH1*, *ETFDH*, *GCDH*, *IVD*, *SCP2*, were upregulated in FO-MFD compared to CLA-MFD, which might suggest that different genes trigger similar biological processes under different MFD conditions. It is probably worth mentioning that considerable effort has been invested in elucidating the relationship between MFD and lipogenesis^9^, but the role of lipid catabolic processes is less known.

Among these upregulated genes in FO-MFD, when compared to CLA-MFD, there was, for instance, activation of enoyl coenzyme A hydratase 1 (*ECH1*). In mouse liver, overexpression of this enzyme reduces lipid accumulation when animals ingest high fat diets and inhibits lipogenic gene expression^26^. Missense mutations in another gene upregulated in FO-MFD that codes for flavoprotein dehydrogenase (*ETFDH*) have been related to glutaric aciduria type IIC in humans. The clinical signs of this disease include, among others, accumulation of subcutaneous fat, liver steatosis and lipid storage myopathy^27^, which relates the abnormal function of this gene to lipid accumulation. Mutations in the *glutaryl-CoA dehydrogenase (GCDH)* gene have also been related to glutaric aciduria, but to type I in this case. Marti-Masso *et al*.^28^ linked alterations in mitochondrial fatty acid metabolism due to mutations in the *GCDH* gene to the development of muscular dystonia in glutaric aciduria type I in humans. Finally, the *SCP2* gene, encoding the sterol carrier protein 2, is increased in the mammary gland of cows supplemented with diets rich in unsaturated fatty acids that elicit MFD^12^.

**Figure 2:**
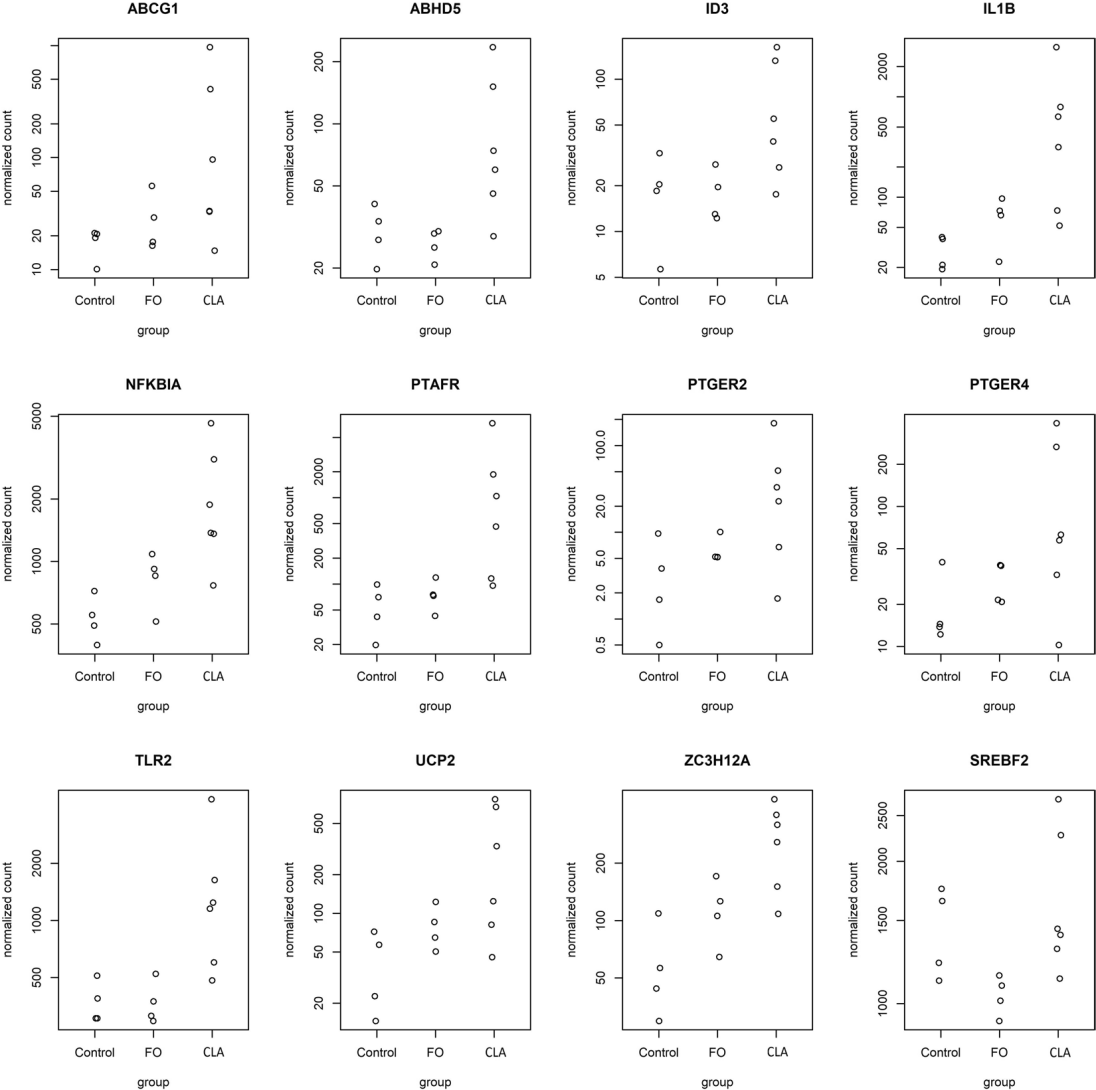
Plot showing gene expression (DESeq2 normalized counts) of eleven genes linked to lipid catabolism and lipid storage found upregulated in CLA-induced milk fat depression (CLA-MFD) compared to control and fish oil-induced MFD (FO-MFD) and *SREBF2* gene (found upregulated when CLA-MFD was compared to FO-MFD).

Among genes upregulated in CLA-MFD compared to FO-MFD, the enriched GO biological process terms related to lipid metabolism were linked to “*lipid storage*”, “*regulation of lipid storage*”, and “*response to fatty acid*”. Eleven genes (*ABCG1*, *ABHD5*, *ID3*, *IL1B*, *NFKBIA*, *PTAFR*, *PTGER2*, *PTGER4*, *TLR2*, *UCP2*, *ZC3H12A*) clustered within these enriched terms were also upregulated in CLA-MFD compared to control. A contrast in the expression levels of these genes for the three considered conditions is presented in Fig. 2, as they may be key factors in determining the specific mechanisms underlying the occurrence of CLA-induced MFD. We are aware of very few reports in ruminants relating variations in the expression of these genes and implications in lipid metabolism. For instance, increased *ABCG1* expression in CLA-MFD samples was detected by qPCR in a previous study^10^ and might suggest changes in cholesterol homeostasis^29^. Concerning the *ABHD5* gene, which encodes the alpha-beta hydrolase domain-containing 5 protein, a key regulator of adipose triglyceride lipase (ATGL)-mediated lipolysis^30^, its upregulation in the adipose tissue of cows supplemented with palmitic acid has been related to enhanced lipolysis to support increased milk yield^31^. The *inhibitor of DNA binding 3 HLH protein* (*ID3*) is involved in the inhibition of FA synthesis in rat adipocytes by reducing expression of fatty acid synthase through inhibition of SREBP-1c^32^. The *PTGER2* and *PTGER4* genes encode for prostaglandin receptors E 2 and 4, respectively, and *PTGER4* knockout mice exhibit a lean phenotype due to the reduction of adipose tissue and the accumulation of triglycerides in other organs due to impaired triglyceride clearance^33^. The *UCP2* gene encodes for the uncoupling protein 2 of the respiratory chain, and its function has been associated with increased energy expenditure^34^. This gene has been demonstrated to be upregulated in mice supplemented with CLA^35–37^ and in the mammary gland of cows under plant oil-induced MFD conditions^12^, which are associated with the anti-lipogenic effect of *trans*-10 *cis*-12 CLA^1^. Finally, *ZC3H12A* encodes the regnase-1 protein and in *Caenorhabditis elegans* has been linked to lipid metabolism by acting as a posttranscriptional regulator^38^. Specifically, regnase-1 protein has been shown to favor fat accumulation by degrading mRNA encoding the ETS-4 fat-loss-promoting transcription factor^38^. In cattle, dietary restriction increased *ZC3H12A* expression in the rumen epithelium and liver, which was speculated to have some association with the concomitant downregulation of genes involved in nutrient processing and metabolism^39,40^. Hence, further research is necessary to identify the potential role of these genes in the mammary gland of dairy ewes suffering from CLA-induced MFD.

Concerning downregulated genes, the highest enriched term when CLA-MFD samples were compared to controls was *“citrate metabolic process*”. Among genes within this term, we found the isocitrate dehydrogenase 1 (*IDH1*), which has been suggested to play a key role in generating the primary source of NADPH necessary for *de novo* fatty acid synthesis in the bovine mammary gland^41^. CLA has been proposed to inhibit mammary lipogenesis, at least to a certain extent, due to the decrease of *IDH1* expression^42^. Interestingly, this gene was also downregulated in animals on the CLA diet when compared to those receiving FO, suggesting differences between these dietary supplements with respect to regulation of this gene. In this regard, changes in milk FA profile would support a greater inhibition of *de novo* synthesis in CLA-MFD treatment than in FO-MFD^10^.

In addition, among the genes downregulated in CLA-MFD compared to control, there were also GO terms related to fatty acid and acyl-CoA metabolic processes. Within these terms, we detected genes encoding key proteins involved in *de novo* fatty acid synthesis, such as fatty acid synthase (*FASN*) and acetyl-CoA carboxylase alpha (*ACACA*)^43^, consistent with the trend observed in qPCR analysis^10^. The latter enzyme was also reported to be downregulated in FO-MFD compared to control^11^. Moreover, other genes related to lipid metabolism were also downregulated in both CLA- and FO-MFD when these conditions were compared to the control, such as some genes associated with activation of acetoacetate to acetoacetyl-CoA (*AACS*), activation of fatty acids with CoA (*ACSS2*, *ACSS3*), and desaturation of fatty acids (*FADS2*)^43^. Expression of most of these lipogenic genes is controlled by the sterol regulatory element-binding protein-1 (SREBP1) signaling pathway^44,45^, which was found to be inhibited in our previous study comparing the transcriptomes of FO-MFD and control ewes^11^. In addition to the differences between the mechanisms of action of CLA and FO, these downregulated genes suggest there are common pathways activated in both types of diet-induced MFD.

Interestingly, the sterol regulatory element-binding transcription factor 2 (*SREBF2*) was downregulated in FO-MFD ewes compared to those receiving CLA (Fig. 2). Although both *SREBF1* and *SREBF2* have been associated with lipid metabolism in the mammary gland^43,46^, each isoform may have different roles^47^. Thus, *SREBF2* would be mainly involved in cholesterol biosynthesis^47^, whereas SRBEP1 signaling would be crucial to MFD^45,48^. As mentioned above, several genes clustered in this pathway were found to be downregulated in CLA and FO, but these did not include *SREBF1*. This lack of variation is consistent with our previous qPCR results^10^ but contrasts with most reports on MFD in ruminants^13,45,48^. The complex posttranscriptional regulation of *SREBF1*^45^, as well as the relatively advanced stage of lactation, may be of relevance in this regard. CLA-induced downregulation of *SREBF1* was previously detected in sheep during early and mid-lactation (15 and 70 days in milk) but not during late lactation (120 days in milk^49^), and our ewes were at approximately 102–103 days in milk when samples were collected.

In summary, we identified several downregulated genes involved in the synthesis of fatty acids, such as *ACACA*, *AACS*, *ACSS2*, *ACSS3* and *FADS2*, in response to both CLA- and FO-MFD conditions. However, although the phenotype induced by dietary supplementation with either CLA or FO is similar^4,10^, inclusion of CLA is related to global expression patterns showing higher variability than the addition of FO. Moreover, CLA causes a significant impact in terms of gene expression changes by influencing not only the expression of genes related to lipid metabolism but also of others involved in different biological processes, especially immunity. Furthermore, some genes related to lipid catabolism were upregulated in CLA-MFD compared to control and FO groups, while others implicated in the same function were upregulated by FO- compared to CLA-MFD. This suggests that both supplements might affect the expression of different genes associated with biological processes leading to similar phenotypes. Finally, this study provides a list of candidate genes that are not usually considered in the nutrigenomics of MFD but that may act as key regulators in biological processes inducing low-fat milk syndrome in dairy ewes fed either marine lipid- or CLA-supplemented diets.

## Supplementary information

Tables S1-S5
